# Efficacy of antepartum administration of hepatitis B immunoglobulin in preventing mother‐to‐child transmission of hepatitis B virus

**DOI:** 10.1111/jvh.13123

**Published:** 2019-06-03

**Authors:** Mengyu Zhao, Huaibin Zou, Yu Chen, Sujun Zheng, Zhongping Duan

**Affiliations:** ^1^ Difficult & Complicated Liver Diseases and Artificial Liver Center, Beijing Youan Hospital Capital Medical University Beijing China; ^2^ Beijing Municipal Key Laboratory of Liver Failure and Artificial Liver Treatment Research Beijing China

**Keywords:** efficacy, hepatitis B immunoglobulin, hepatitis B virus, mother‐to‐child transmission, pregnancy

## Abstract

The aim of this study was to investigate the efficacy of antepartum administration of three doses of hepatitis B immunoglobulin (HBIG) in interrupting mother‐to‐child transmission (MTCT) of hepatitis B virus (HBV). In this trial, a total of 728 HBeAg‐positive pregnant women with chronic HBV infection who had an HBV DNA level higher than 6log_10_ copies/mL were enrolled. They were divided into three groups based on individual preference. Subjects in group A and group B received 200 IU (unit) HBIG and 400 IU (unit) HBIG intramuscularly once a month at the third, second and first month before delivery, respectively. Subjects in the control group (C) received no special treatment. All the infants received passive‐active immunoprophylaxis. The HBsAg‐positive rate of all infants at 7‐12 months of age was 5.1% (37/728). Specifically, the HBsAg‐positive rate of infants was comparable in all three groups (5.3% vs 5.1% vs 5%, *P* = 0.988). No significant difference was found in anti‐HBs levels between the infants aged 7‐12 months in the three groups (*P* = 0.469). HBV DNA levels of the umbilical cord blood in the HBV‐infected group were higher than those in the uninfected group (5.2 vs 3.4log_10_ copies/mL, *P < *0.001), and these with family history of HBV infection were also higher (45.9% vs 28.5%, *P* = 0.034). To conclude, administration of passive‐active immunoprophylaxis to infants contributed to effective prevention of the MTCT of HBV; extra antepartum administration of HBIG during pregnancy could not decrease the rate of MTCT or increase the anti‐HBs levels of infants born to HBsAg‐positive mothers with HBV DNA higher than 6log_10_ copies/mL.

**Abbreviations**
ALB: albumin
ALT: alanine aminotransferase
AST: aspartate transaminase
CHB: chronic hepatitis B
CHE: cholinesterase
CHOL: cholesterol
GLU: blood glucose
HGB: haemoglobin
HBIG: hepatitis B immunoglobulin
HBV: hepatitis B virus
MTCT: mother‐to‐child transmission
PLT: platelet
SD: standard deviation
TBIL: total bilirubin
TDF: tenofovir disoproxil fumarate

## 1 INTRODUCTION

Hepatitis B virus (HBV) infection is still one of the most prominent public health problems in China. Mother‐to‐child transmission (MTCT) is considered as the main transmission route of chronic HBV infection. Fifty per cent of infants born to hepatitis B surface antigen (HBsAg)‐positive mothers will become carriers without immunization.^1^ Hence, the prevention of MTCT is essential in reducing the global burden of chronic HBV infection.^2^, ^3^, ^4^, ^5^

In the 1980s, studies showed that in newborns of HBsAg‐positive mothers, the MTCT rate was reduced to 23% after vaccination without HBIG^6^ and to 3%‐7% after passive‐active immunization.^7^ In 2010, the chronic hepatitis B (CHB) guidelines of China suggested that infants born to HBsAg‐positive mothers should be administrated passive‐active immunization by injecting HBIG within 24 hours after birth (preferably within 12 hours) and 10 μg HB vaccine at a different anatomical site.^8^ In addition, 10 μg of the HB vaccine was administered to infants at 1 and 6 months of age. But, 5%‐10% of newborns born to HBsAg‐positive mothers can still be chronically infected with HBV.^9^ It has been recognized that pregnant women with high viral load are the reason for passive‐active immunization failure.^10^ Therefore, more and more scholars recommend application of antiviral drugs in the third trimester to block MTCT in pregnant women with high HBV DNA load.^11^, ^12^ However, it is still far from ideal in completely eradicating MTCT of HBV due to intrauterine infection and immunoprophylaxis failure in a small number of individuals.^13^

HBIG is a purified solution of human immunoglobulin with high titres of antibody against hepatitis B surface antigen (anti‐HBs). It is derived from plasma donated by individuals immune to HBV infection.^14^ Early reports showed that the antepartum administration of HBIG during pregnancy decreased the HBV DNA levels of mothers and the MTCT rates.^15^, ^16^ However, recent reports suggested that antepartum administration of HBIG failed to improve the efficacy of preventing MTCT of HBV. Thus, the efficacy of antepartum administration of HBIG during pregnancy in preventing MTCT needs to be confirmed by further studies.

## 2 MATERIALS AND METHODS

### 2.1 Patients

In this trial, all of the 728 HBsAg‐positive pregnant women were reviewed in a hospital clinic for routine antenatal care in the Department of Obstetrics and Gynecology in Beijing Youan Hospital, Capital Medical University, from 2014 to 2018.

The eligible criteria included the following: (a) HBsAg, HBeAg‐positive; (b) 20‐40 years old; (c) HBV DNA level higher than 6log_10_ copies/mL; and (d) participants willing to provide written informed consent and adhere to the trial protocol. Chronic HBV infection was defined as HBsAg seropositive status and/or HBV DNA positivity at 6 months or beyond. The diagnostic criteria for HBV‐positive patients were based on the Guideline of Prevention and Treatment for Chronic Hepatitis B (2015 Update).^8^

The exclusion criteria were as follows: coinfection with human immunodeficiency virus (HIV) type 1, hepatitis C virus, or hepatitis delta virus; a history of congenital malformation in a previous pregnancy; evidence of hepatocellular carcinoma or liver decompensation; clinical signs of threatened miscarriage; and ultrasonographic evidence of foetal deformity.

The trial was performed in accordance with the International Conference on Harmonisation Good Clinical Practice guidelines and the Declaration of Helsinki.

### 2.2 Methods

A total of 728 pregnant women were divided into three groups and administered different doses of antepartum HBIG based on individual preference. Group A: 256 mothers received 200 IU (unit) HBIG intramuscularly once a month at the third, second and first month before delivery; group B: 302 mothers received 400 IU (unit) HBIG once a month at the third, second and first month before delivery; the control group (C): 170 mothers were given no special treatment. All infants received passive‐active immunoprophylaxis that received HBIG at birth and hepatitis B vaccine at birth and at 1 and 6 months.

### 2.3 Laboratory methods

Maternal HBV DNA was tested before delivery; HBsAg, anti‐HBs, hepatitis B e antigen (HBeAg), anti‐HBe, anti‐HBc and HBV DNA of infants were tested at the age of 7‐12 months. For HBsAg‐positive mothers and their infants, alanine aminotransferase (ALT), aspartate transaminase (AST), total bilirubin (TBIL), blood glucose (GLU), cholinesterase (CHE) and cholesterol (CHOL) were measured using an Olympus Automatic Biochemical Analyzer AU5400 (Olympus). HBV seromarkers were performed by chemiluminescence methods. The level of HBV DNA was examined using the Cobas HBV Amplicor Monitor assay (Roche Diagnostics). Nationality of Han was the major ethnic group in China.

### 2.4 Outcome assessment

HBsAg positivity of infants at the age of 7‐12 months was considered to be immunoprophylaxis failure and HBV infection. Infants were defined as hepatitis B positive if HBsAg ≥ 0.05 IU/mL or HBV DNA ≥ 20 IU/mL. Immunity was defined as anti‐HBs‐positive ≥ 10 IU/mL.

### 2.5 Statistical analysis

The data were analysed using Statistical Package for Social Science (SPSS) for windows, version 23.0. Continuous variables conforming to the normal distribution were characterized by the mean and standard deviation (SD). The three groups were compared and analysed by one‐way ANOVA. The comparison between the two groups was analysed by Student's *t* test. The characteristics of the continuous variables not consistent with the normal distribution were described using the median (25% to 75% IQR). The comparison between the three groups and the two groups were analysed by Kruskal‐Wallis H and Mann‐Whitney *U* test. Categorical variables were characterized by the proportion and analysed using the chi‐squared (*χ* ^2^) test or Fisher's exact tests. Statistical significance was defined as *P* < 0.05.

## 3 RESULTS

### 3.1 Demographic characteristics

A total of 728 mother‐infant pairs were enrolled in this study, with maternal age 27.4 ± 4.2 years old. 96.2% (700/728) of mothers were Han. The HBsAg‐positive rate of infants aged 7‐12 months was 5.1% (37/728). The mothers who received no special treatment, 200IU HBIG injection and 400IU HBIG injection accounted for 23.4% (170/728), 35.2% (256/728) and 41.4% (302/728), respectively. Baseline characteristics of mothers and infants stratified by different doses of HBIG injection were comparable (*P* > 0.05, Table 1).

**Table 1:** Baseline characteristics of the participants

Characteristics	Control group (n = 170)	Group A (n = 256)	Group B (n = 302)	*P* value
Maternal data				
Age (y, mean ± SD)	28.0 ± 4.4	27.5 ± 4.2	27 ± 3.9	0.053
Nationality of Han	161 (94.7%)	249 (97.3%)	290 (96.0%)	0.400
Family history	52 (30.6%)	80 (31.3%)	82 (27.2%)	0.789
Delivery modes				
Caesarean section	93 (54.7%)	132 (51.6%)	137 (45.4%)	0.112
Spontaneous labour	76 (44.7%)	124 (48.4%)	164 (54.3%)	
Infant data				
Sex (M/F）	93/76	144/112	161/141	0.432
Nationality of Han	162 (95.3%)	244 (95.3%)	289 (95.7%)	0.681
Intrauterine distress	35 (20.6%)	41 (16.0%)	43 (14.2%)	0.174
Amniotic fluid dyeing	91 (53.5%)	194 (75.8%)	248 (82.1%)	0.085
Birth weight (g, mean ± SD)	3370.9 ± 452.8	3327.1 ± 544.3	3370.5 ± 435.0	0.505
Apgar (score, mean ± SD)				
1 min	9.4 ± 0.8	9.4 ± 0.8	9.4 ± 0.7	0.829
5 min	10 ± 0.1	10 ± 0.2	10 ± 0.1	0.991
10 min	10.0	10 ± 0.1	10 ± 0.1	0.639

Note

Group A: mothers received 200 IU (unit) HBIG intramuscularly, group B: mothers received 400 IU (unit) HBIG, and the control group mothers were given no special treatment. *P* values were calculated between groups by using analysis of variance for continuous variables and the *χ* ^2 ^test for categorical variables.

Abbreviation: SD, standard deviation.

### 3.2 The outcomes of different doses of HBIG injection on mothers

Based on the comparison between the HBV DNA levels of mothers injected with different doses of HBIG before delivery, antepartum administration of HBIG was found incapable of decreasing the HBV DNA levels (7.1 vs 7.3 vs 7.4log_10_ copies/mL, *P* = 0.555). AST, TBIL, GLU, HGB, PLT, CHE and CHOL of participants obtained in the three groups were comparable (*P* > 0.05, Table 2).

**Table 2:** The effects of antepartum administration of HBIG on mothers

Characteristics	Control group (n = 170)	Group A (n = 256)	Group B (n = 302)	*P* value
ALT (U/L)	16.6 (11.7, 21.4)	13.7 (10.7, 19.5)	15.4 (11.3, 21.7）	0.023
AST (U/L)	22.9 (18.8, 30.2)	22.0 (18.5, 28.0)	23.3 (19.2, 30.5)	0.279
TBIL (mmol/L, mean ± SD)	11.0 ± 4.6	11.7 ± 5.4	11.7 ± 6.6	0.428
GLU (mmol/L, mean ± SD)	4.7 ± 3.3	4.4 ± 2.6	4.5 ± 2.6	0.681
ALB (g/L, mean ± SD)	32.9 ± 4.0	32.0 ± 3.6	32.0 ± 3.5	0.018
HGB (g/L, mean ± SD)	112.3 ± 16.7	112.8 ± 12.8	111.2 ± 14.2	0.432
PLT (×10^9^/L, mean ± SD)	204.7 ± 56.9	198.3 ± 52.1	201.3 ± 52.4	0.487
CHE (mmol/L, mean ± SD)	5282 (4415, 6150）	4949 (4261, 5933）	5149 (4283, 6294）	0.096
CHOL (mmol/L, mean ± SD)	5.7 ± 1.2	5.5 ± 1.2	5.9 ± 3.1	0.133
HBV DNA levels (log_10_ copies/mL, mean ± SD)^a^	7.1 ± 0.7	7.3 ± 0.6	7.4 ± 0.5	0.555

Abbreviations: ALB, albumin; ALT, alanine aminotransferase; AST, aspartate aminotransferase; CHE, cholinesterase; CHOL, cholesterol; GLU, blood glucose; HGB, haemoglobin; PLT, platelet; TBIL, total bilirubin.

a The maternal HBV DNA was measured as log_10_ copies/Ml.

ALB between the control group, group A and group B exhibited statistically significant differences (*P* = 0.018). The control group had higher ALB than groups A and B (*P* = 0.011, *P* = 0.01). No significant difference in ALB was found between group A and group B (*P* > 0.05). Similarly, there were statistically significant differences in ALT levels between the three groups (*P* = 0.023), with ALT levels in group A lower than those in the control group and group B (*P* = 0.013, *P* = 0.027). No significant difference in ALT was found between the control group and group B (*P* > 0.05, Table 2). The levels of ALT and ALB in the three groups were within normal limits, so there was no clinical significance.

### 3.3 The effects of infants born to mothers injected with different doses of HBIG

**Figure 1:**
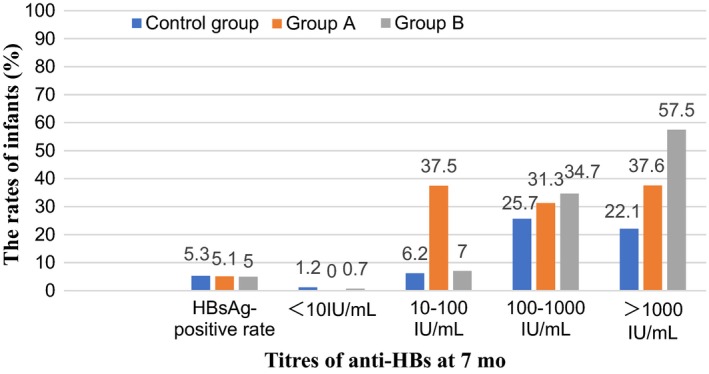
The outcomes of infants born to mothers injected with different doses of HBIG

The HBsAg‐positive rate of infants aged 7‐12 months between the three groups were comparable (5.3% vs 5.1% vs 5%, *P* = 0.988). No significant difference was found in the age of mothers whose infants had different anti‐HBs levels (*P* = 0.633, Table 3). The anti‐HBs‐positive rate of infants at the age of 7‐12 months who were born to mothers administrated 200 IU HBIG before delivery reached 100%. Although there were no significant differences in anti‐HBs levels between infants aged 7‐12 months in the three groups (*P* = 0.469, Figure 1), the proportion of infants in 100‐1000 IU/mL and >1000 IU/mL rose as the dose of maternal injection of HBIG increased. Infants with anti‐HBs levels of 100‐1000 IU/mL in the control group, group A and group B accounted for 25.7%, 31.3% and 34.7%, respectively. Those infants in the three groups with anti‐HBs levels of >1000 IU/mL accounted for 90(22.1%), 153(37.6%) and 164(57.5%), respectively.

**Table 3:** Age of mothers according to infant anti‐HBs levels

Anti‐HBs levels	Age (y, mean ± SD)	*P* value
<10 IU/mL	26.9 ± 4.4	0.633
10‐100 IU/mL	27.0 ± 5.0	
100‐1000 IU/mL	27.6 ± 4.0	
>1000 IU/mL	27.4 ± 4.1	

### 3.4 Association of different clinical characteristics with HBV infection in infants

The maternal HBV DNA levels before delivery in the HBV‐infected group and the uninfected group were comparable (7.5 vs 7.3log_10_ copies/mL, *P* = 0.176). A significant difference of HBV DNA levels was found in umbilical cord blood of the HBV‐infected group and the uninfected group (5.2 vs 3.4log_10_ copies/mL, *P* < 0.001). The two groups exhibited no significant differences in age, delivery modes, AST, TBIL, GLU, HGB, PLT, CHE, CHOL, intrauterine distress, amniotic fluid dyeing or Apgar score at 1 minute, 5 minutes and 10 minutes (*P* > 0.05, Table 4). Infants born to mothers with HBV family history were more likely to be infected by HBV (45.9% vs 28.5%, *P* = 0.034). HBV family history was defined as first‐degree relatives of pregnant women who had chronic HBV infection. Infants in the uninfected group achieved higher birth weight than those in the HBV‐infected group (*P* = 0.02). There was no significant difference in HBV infection status of infants born to mothers injected with different doses of HBIG (*P* = 0,988, Table 5).

**Table 4:** Clinical characteristics of infants and mothers stratified by HBV infection status

Characteristics	HBV‐infected group (n = 37)	Uninfected group (n = 691)	*P* value
Maternal data			
Age (y, mean ± SD)	26.2 ± 4.6	27.5 ± 4.1	0.079
Nationality of Han	35 (95.6%)	665 (96.2%)	0.613
Family history	17 (45.9%)	197 (28.5%)	0.034
Delivery modes			
Caesarean section	15 (40.5%)	347 (50.2%)	0.244
Spontaneous labour	22 (59.5%)	342 (49.5%)	
ALT (U/L)	13.8 (11.1, 18.6)	15.0 (11.0, 20.9)	0.302
AST (U/L)	21.8 (17.4, 29.8)	22.8 (18.8, 29.2)	0.327
TBIL (mmol/L, mean ± SD)	11.6 ± 4.4	11.5 ± 5.8	0.931
GLU (mmol/L, mean ± SD)	4.3 ± 0.7	4.5 ± 2.9	0.707
ALB (g/L, mean ± SD)	33.0 ± 4.0	32.1 ± 3.6	0.146
HGB (g/L, mean ± SD)	111.3 ± 13.3	112.1 ± 14.4	0.758
PLT (×10^9^/L, mean ± SD)	207.8 ± 49.3	200.7 ± 53.6	0.427
CHE (mmol/L, mean ± SD)	4950 (4592.5, 6352.5)	5081 (4290.5, 6118）	0.603
CHOL (mmol/L, mean ± SD)	5.9 ± 1.3	5.7 ± 2.2	0.604
HBV DNA levels (log_10_ copies/mL, mean ± SD)	7.5 ± 0.5	7.3 ± 0.6	0.176
Infant data			
Sex (M/F)	19/18	379/312	0.885
Nationality of Han	34 (91.9%)	661 (95.7%)	0.213
Intrauterine distress	7 (18.9%)	112 (16.2%)	0.579
Amniotic fluid dyeing	27 (73.0%)	506 (73.2%)	0.967
Birth weight (g, mean ± SD)	3123.0 ± 611.9	3167.8 ± 469.2	0.02
Apgar (score, mean ± SD)			
1 min	9.2 ± 0.9	9.4 ± 0.8	0.123
5 min	10 ± 0.2	10 ± 0.2	0.548
10 min	10.0	10 ± 0.1	0.7
HBV DNA levels in cord blood (mean ± SD)	5.2 ± 1.7	3.4 ± 0.8	<0.001

Abbreviations: ALB, albumin; ALT, alanine aminotransferase; AST, aspartate aminotransferase; CHE, cholinesterase; CHOL, cholesterol; GLU, blood glucose; HGB, haemoglobin; PLT, platelet; TBIL, total bilirubin.

**Table 5:** HBV infection status of infants born to mothers injected with different doses of HBIG

Characteristics	Infection (n = 37)	Noninfection (n = 691)	*P* value
HBIG injection			
Noninjection	9 (24.3%)	161 (23.3%)	0.988
200 IU	13 (35.1%)	243 (35.2%)	
400 IU	15 (40.5%)	287 (41.5%)	

## 4 DISCUSSION

Passive‐active immunization is conducive to effective decrease in the rate of MTCT to 5%‐10%. However, pregnant women with high viral load are associated with passive‐active immunization failure.^10^ Nucleoside/nucleotide analogs were useful and relatively safe in reducing the incidence of MTCT in pregnant women with high HBV DNA load.^11^, ^12^ Pan et al^17^ enrolled 200 HBeAg‐positive mothers with HBV DNA level higher than 200 000 IU/mL who received the usual care without antiviral therapy or tenofovir disoproxil fumarate (TDF) (at an oral dose of 300 mg per day) from 30 to 32 weeks of gestation until postpartum week 4; they found the rate of MTCT was lower in the TDF group than in those without antiviral therapy. Jourdain et al^18^ also found that none of the 147 infants (0%) born to mothers who received TDF in the third trimester were infected. However, the efficacy of preventing MTCT could not reach 100%. Once the infection is already established, it is too late to stimulate the production of anti‐HBs by the HB vaccine.

Some experts recommend that HBsAg‐positive pregnant women receive small dosages of HBIG in their third trimester of pregnancy to interrupt HBV intrauterine infection.^16^, ^19^ In theory, antepartum administration of HBIG during pregnancy can reduce the rate of MTCT. The possible mechanism is described below: (a) After 20 weeks of gestation, placental trophoblast cells have the function of actively transferring IgG‐type antibodies to the foetus. (b) HBIG regulates the immunodeficiency state caused by HBV infection. It promotes the secretion of interferon‐γ and interleukin‐12 by increasing the activation of Th1 cells, which is beneficial to the clearance of HBV in pregnant women as well as to the reduction of HBV DNA in vivo. (c) HBIG binds to HBV, activating the complement system, increasing humoural immunity and rapidly clearing HBV.

In our study, antepartum administration of HBIG did not prevent MTCT of HBV. The HBsAg‐positive rate of all the infants aged 7‐12 months was 5.1% (37/728). There were no significant differences in HBsAg‐positive rates between the control group and the 200 IU and 400 IU groups (5.3%, 5.1%, 5%, *P* = 0.988). A study conducted by Zhang et al included 224 mothers who received antepartum administration of HBIG once a month in late pregnancy. They also found no significant difference in HBV infection rate of infants between the groups of mothers with antepartum administration of HBIG and without HBIG (4.5% vs 3.1%, *P* = 0.293).^20^ This was in line with previous findings suggesting that injecting HBIG in HBsAg‐positive pregnant women during pregnancy was not effective in preventing MTCT of HBV.^21^, ^22^ Yuan et al was performed a study on 250 HBeAg‐positive pregnant women who were randomly divided into study (117 cases, received HBIG 400 IU intramuscularly once a month at the third, second and first month before delivery) and control groups (133 cases, no antepartum treatment). No significant difference was found in the proportion of HBsAg‐positive infants between the two groups at one year of age (11.2% vs 12.78%, *P* > 0.05).^23^ After analysing the cost‐effectiveness of different methods, some scholars found that passive‐active immunization on infants could reduce the rates of CHB infection in children at the lowest cost, without the necessity to inject HBIG to mothers during pregnancy.^24^

The reason for the negative result may be that the small dosage of HBIG injection (200 IU or 400 IU) did not decrease maternal HBV load and was insufficient for HBIG to enter the foetal circulation. In theory, complete neutralization of HBsAg by HBIG in vitro was possible, and 50% inhibition with HBsAg levels of 68 and 120 ng/mL was achieved with concentrations between 100 and 250 IU/L of HBIG. HBsAg‐positive pregnant women cannot receive large doses of HBIG during pregnancy in China because massive HBIG injection may cause HBV mutation.^25^ This may subsequently result in the failure of passive‐active immunoprophylaxis and lead to increased resistance of mutated virus to antiviral agents.^20^, ^26^ Furthermore, in vivo neutralization of HBsAg by HBIG was achieved only in patients with low HBsAg levels.^27^, ^28^ In this case, the dose of antepartum HBIG administered to mothers was not enough to block MTCT of HBV. Moreover, the mean half‐life of HBIG was reported to be 24.0 ± 3.8 days.^29^, ^30^ By the time infants received the third dose of vaccine (6 months), the HBIG administered at birth would have been cleared. If HBV DNA is actively replicating, the neutralization efficacy of HBIG would be limited and transitory. Thus, the third vaccination may play an important role in the development of anti‐HBs in infants.

In the present study, although the anti‐HBs‐positive rate of infants born to mothers injected with 200 IU HBIG reached 100%, there were no significant differences between the three groups regarding anti‐HBs levels in <10 IU/mL, 10‐100 IU/mL, 100‐1000 IU/mL or >1000 IU/mL group (*P* = 0.469). As a result, anti‐HBs levels were found to have no relationship with the injection of HBIG among mothers and the dose of administration of HBIG. This is consistent with several studies. Xiao et al reported the efficacy of HBIG in the interruption of HBV infection, but found no significant increase in the newborn anti‐HBs seropositivity rate, either.^31^ The study by Han et al^32^ displayed no significant decrease in maternal HBV DNA load, and none of their newborns had anti‐HBs.

In the present study, the maternal HBV DNA levels before delivery in the HBV‐infected group and the uninfected group were comparable (7.5 vs 7.3log_10_ copies/mL, *P* = 0.176); however, significant differences of HBV DNA levels were found in umbilical cord blood between the HBV‐infected and the uninfected groups (5.2 vs 3.4log_10_ copies/mL, *P* < 0.001). The results are consistent with some previous studies. Zou et al^10^ concluded that detectable HBV DNA in the cord blood (OR = 39.67, 95% CI: 14.22‐110.64) is an independent risk factor for immunoprophylaxis failure. Studies mentioned above suggest that the anti‐HBs levels of mothers may greatly decrease passing through the placental barrier, resulting in lower anti‐HBs titres in the infants. Therefore, the mechanism of placental barrier in preventing MTCT of HBV should be examined by further studies, in which additional factors such as genetic factors, quality of vaccine or other causes can be considered.

Although there were statistically significant differences in ALT and ALB levels between the three groups, all the indicators were within the normal limits, so the clinical significance was considered to be small. In addition, HBV infection rates of infants born to mothers with family history were higher than those without family history (45.9% vs 28.5%, *P* = 0.034). We consider that blocking failure may be related to genetic sequences. Some studies have found that there is aggregation of nonresponders in nonresponse family members. The positive rate and average levels of anti‐HBs of the first‐degree relatives in nonresponders/low responders were significantly lower than those in high responders after three doses of the hepatitis B vaccine, indicating that the response ability to the hepatitis B vaccine of first‐degree relatives in nonresponders/low responders was lower than that in strong responders, indicating that nonresponse/low response is related to the individual's genetic background. Studies have also shown that ‘S’ gene mutations cause immune escape, leading to vaccine immune failure.^33^ McDermott et al^34^ confirmed that DRBl*0701 and DQBl*02 were closely related to no response after vaccination. In addition, DRl4‐DR52 was also associated with a low response to hepatitis B vaccine.^35^

To conclude, administration of passive‐active immunoprophylaxis to infants contributed to effective prevention of the MTCT of HBV; extra antepartum administration of HBIG during pregnancy could not decrease the rate of MTCT or increase the anti‐HBs levels of infants born to HBsAg‐positive mothers with HBV DNA higher than 6log_10_ copies/mL.

## CONFLICT OF INTEREST

The authors do not have any conflict of interests to report.

## References

[1] Chien YC , Jan CF , Kuo HS , et al. Nationwide hepatitis B vaccination program in Taiwan: effectiveness in the 20 years after it was launched. Epidemiol Rev. 2006;28:126‐135. 16782778 10.1093/epirev/mxj010

[2] Visvanathan K , Dusheiko G , Giles M , et al. Managing HBV in pregnancy. Prevention, prophylaxis, treatment and follow‐up: position paper produced by Australian, UK and New Zealand key opinion leaders. Gut. 2016;65(2):340‐350. 26475631 10.1136/gutjnl-2015-310317

[3] Yi P , Chen R , Huang Y , Zhou R‐R , Fan X‐G . Management of mother‐to‐child transmission of hepatitis B virus: Propositions and challenges. J Clin Virol. 2016;77:32‐39. 26895227 10.1016/j.jcv.2016.02.003

[4] Lok AS . Prevention of hepatitis B virus‐related hepatocellular carcinoma. Gastroenterology. 2004;127(5 Suppl 1):S303‐309. 15508098 10.1053/j.gastro.2004.09.045

[5] Lozano R , Naghavi M , Foreman K , et al. Global and regional mortality from 235 causes of death for 20 age groups in 1990 and 2010: a systematic analysis for the Global Burden of Disease Study 2010. Lancet. 2012;380(9859):2095‐2128. 23245604 10.1016/S0140-6736(12)61728-0 PMC10790329

[6] Beasley RP , Hwang LY , Lin CC , et al. Hepatitis B immune globulin (HBIG) efficacy in the interruption of perinatal transmission of hepatitis B virus carrier state. Initial report of a randomised double‐blind placebo‐controlled trial. Lancet. 1981; 2(8243): 388‐393. 6115159 10.1016/s0140-6736(81)90832-1

[7] Wong VivianCW , Reesink HenkW , Ip HenriettaMH , et al. Prevention of the HBsAg carrier state in newborn infants of mothers who are chronic carriers of HBsAg and HBeAg by administration of hepatitis‐B vaccine and hepatitis‐B immunoglobulin. Double‐blind randomised placebo‐controlled study . Lancet. 1984;1(8383):921‐926. 6143868 10.1016/s0140-6736(84)92388-2

[8] Hou J , Wang G , Wang F , et al. Guideline of prevention and treatment for chronic hepatitis B (2015 Update). J Clin Transl Hepatol. 2017;5(4):297‐318. 29226097 10.14218/JCTH.2016.00019 PMC5719188

[9] Pan CQ , Duan Z , Bhamidimarri KR , et al. An algorithm for risk assessment and intervention of mother to child transmission of hepatitis B virus. Clin Gastroenterol Hepatol. 2012;10(5):452‐459. 22079509 10.1016/j.cgh.2011.10.041

[10] Zou H , Chen Y , Duan Z , Zhang H , Pan C . Virologic factors associated with failure to passive‐active immunoprophylaxis in infants born to HBsAg‐positive mothers. J Viral Hepat. 2012;19(2):e18‐e25. 22239517 10.1111/j.1365-2893.2011.01492.x

[11] Goyal A , Murray JM . The impact of vaccination and antiviral therapy on hepatitis B and hepatitis D epidemiology. PLoS ONE. 2014;9(10):e110143. 25313681 10.1371/journal.pone.0110143 PMC4196970

[12] Cheung KW , Seto MT , Wong SF . Towards complete eradication of hepatitis B infection from perinatal transmission: review of the mechanisms of in utero infection and the use of antiviral treatment during pregnancy. Eur J Obstet Gynecol Reprod Biol. 2013;169(1):17‐23. 23465469 10.1016/j.ejogrb.2013.02.001

[13] Ma L , Alla NR , Li X , Mynbaev OA , Shi Z . Mother‐to‐child transmission of HBV: review of current clinical management and prevention strategies. Rev Med Virol. 2014;24(6):396‐406. 24956038 10.1002/rmv.1801

[14] Habib S , Shaikh OS . Hepatitis B immune globulin. Drugs Today (Barc). 2007;43(6):379‐394. 17612709 10.1358/dot.2007.43.6.1050792

[15] Zhu Q , Yu G , Yu H , et al. A randomized control trial on interruption of HBV transmission in uterus. Chin Med J (Engl). 2003;116(5):685‐687. 12875680

[16] Li XM , Shi MF , Yang YB , et al. Effect of hepatitis B immunoglobulin on interruption of HBV intrauterine infection. World J Gastroenterol. 2004;10(21):3215‐3217. 15457579 10.3748/wjg.v10.i21.3215 PMC4611277

[17] Pan CQ , Duan Z , Dai E , et al. Tenofovir to prevent hepatitis B transmission in mothers with high viral load. N Engl J Med. 2016;374(24):2324‐2334. 27305192 10.1056/NEJMoa1508660

[18] Jourdain G , Ngo‐Giang‐Huong N , Harrison L , et al. Tenofovir versus placebo to prevent perinatal transmission of hepatitis B. N Engl J Med. 2018;378(10):911‐923. 29514030 10.1056/NEJMoa1708131 PMC5895092

[19] Yue Y , Yang X , Zhang S . Prevention of intrauterine infection by hepatitis B virus with hepatitis B immune globulin: efficacy and mechanism. Chin Med J (Engl). 1999;112(1):37‐39. 11593638

[20] Zhang L , Gui X‐E , Teter C , et al. Effects of hepatitis B immunization on prevention of mother‐to‐infant transmission of hepatitis B virus and on the immune response of infants towards hepatitis B vaccine. Vaccine. 2014;32(46):6091‐6097. 25240752 10.1016/j.vaccine.2014.08.078

[21] Kubo AI , Shlager L , Marks AR , et al. Prevention of vertical transmission of hepatitis B: an observational study. Ann Intern Med. 2014;160(12):828‐835. 24862434 10.7326/M13-2529 PMC4086733

[22] Chen H , Lin L , Hu F , et al. Effects of maternal screening and universal immunization to prevent mother‐to‐infant transmission of HBV. Gastroenterology. 2012;142(4):773‐781.e2. 22198276 10.1053/j.gastro.2011.12.035

[23] Shao ZJ , Li JH , Xu DZ , Jiang QW , Xu JQ . Association of administration of hepatitis B immunoglobulin during pregnancy with intrauterine infection of HBV. Chin J Public Health. 2008;24:135‐136.

[24] Guo Y , Zhang W , Zhang YU , et al. Cost‐effectiveness analysis of preventing mother‐to‐child transmission of hepatitis B by injecting hepatitis B immune globulin. Eur J Gastroenterol Hepatol. 2012;24(12):1363‐1369. 22922527 10.1097/MEG.0b013e32835847c6

[25] Jin H , Zhao Y , Tan Z , et al. Immunization interventions to interrupt hepatitis B virus mother‐to‐child transmission: a meta‐analysis of randomized controlled trials. BMC Pediatr. 2014;14:307. 25526664 10.1186/s12887-014-0307-2 PMC4297423

[26] Yuan J , Lin J , Xu A , et al. Antepartum immunoprophylaxis of three doses of hepatitis B immunoglobulin is not effective: a single‐centre randomized study. J Viral Hepat. 2006;13(9):597‐604. 16907846 10.1111/j.1365-2893.2006.00738.x

[27] Heijtink RA , van Nunen AB , van Bergen P , Östberg L , Osterhaus A , de Man RA . Administration of a human monoclonal antibody (TUVIRUMAB) to chronic hepatitis B patients pre‐treated with lamivudine: monitoring of serum TUVIRUMAB in immune complexes. J Med Virol. 2001;64(4):427‐434. 11468726 10.1002/jmv.1068

[28] van Nunen AB , de Man RA , Heijtink RA , Vossen Actm , Schalm SW . Passive immunization of chronic hepatitis B patients on lamivudine therapy: a feasible issue? J Viral Hepat. 2002;9(3):221‐228. 12010511 10.1046/j.1365-2893.2002.00337.x

[29] Wu Q , Zhuang G‐H , Wang X‐L , Wang L‐R , Li NA , Zhang M . Antibody levels and immune memory 23 years after primary plasma‐derived hepatitis B vaccination: results of a randomized placebo‐controlled trial cohort from China where endemicity is high. Vaccine. 2011;29(12):2302‐2307. 21277403 10.1016/j.vaccine.2011.01.025

[30] Wu Q , Zhuang GH , Wang XL , et al. Comparison of long‐term immunogenicity (23 years) of 10 mug and 20 mug doses of hepatitis B vaccine in healthy children. Hum Vaccin Immunother. 2012;8(8):1071‐1076. 22854666 10.4161/hv.20656 PMC3551878

[31] Xiao XM , Li AZ , Chen X , Zhu YK , Miao J . Prevention of vertical hepatitis B transmission by hepatitis B immunoglobulin in the third trimester of pregnancy. Int J Gynaecol Obstet. 2007;96(3):167‐170. 17296201 10.1016/j.ijgo.2006.11.011

[32] Han ZH , Zhong LH , Wang J , et al. The impact of antepartum injection of hepatitis B immunoglobulin on maternal serum HBV DNA and anti‐HBs in the newborns. Zhonghua Nei Ke Za Zhi. 2007;46(5):376‐378. 17637304

[33] Singh AE , Plitt SS , Osiowy C , et al. Factors associated with vaccine failure and vertical transmission of hepatitis B among a cohort of Canadian mothers and infants. J Viral Hepat. 2011;18(7):468‐473. 20546502 10.1111/j.1365-2893.2010.01333.x

[34] McDermott AB , Zuckerman JN , Sabin CA , Marsh SGE , Madrigal JA . Contribution of human leukocyte antigens to the antibody response to hepatitis B vaccination. Tissue Antigens. 1997;50(1):8‐14. 9243749 10.1111/j.1399-0039.1997.tb02827.x

[35] Hsu H‐Y , Chang M‐H , Ho H‐N , et al. Association of HLA‐DR14‐DR52 with low responsiveness to hepatitis B vaccine in Chinese residents in Taiwan. Vaccine. 1993;11(14):1437‐1440. 8310763 10.1016/0264-410x(93)90173-u

